# Updated Austrian treatment algorithm in HER2+ metastatic breast cancer

**DOI:** 10.1007/s00508-021-01987-9

**Published:** 2022-01-28

**Authors:** Rupert Bartsch, Simon Peter Gampenrieder, Gabriel Rinnerthaler, Edgar Petru, Daniel Egle, Andreas Petzer, Marija Balic, Ursula Pluschnig, Thamer Sliwa, Christian Singer

**Affiliations:** 1 Department of Medicine I, Division of Oncology, Medical University of Vienna, Währinger Gürtel 18–20, 1090 Vienna, Austria; 2 Third Medical Department with Hematology and Medical Oncology, Hemostaseology, Rheumatology and Infectious Diseases, Oncologic Center, Paracelsus Medical University Salzburg, Müllner Hauptstraße 48, 5020 Salzburg, Austria; 3 University Hospital for Gynecology and Obstetrics, Clinical Department of Gynecology, Medical University of Graz, Auenbruggerplatz 14, 8036 Graz, Austria; 4 Department of Gynecology, Breast Cancer Center Tirol, Medical University of Innsbruck, Anichstraße 35, 6020 Innsbruck, Austria; 5 Barmherzige Schwestern, Elisabethinen, Department of Internal Medicine I for Hematology with Stem Cell Transplantation, Hemostaseology and Medical Oncology, Ordensklinikum Linz GmbH, Seilerstätte 4, 4010 Linz, Austria; 6 Department of Internal Medicine, Division of Clinical Oncology, Medical University of Graz, Auenbruggerplatz 15, 8036 Graz, Austria; 7 Department of Internal Medicine and Hematology and Internal Oncology, Klagenfurt Hospital, Feschnigstraße 11, 9020 Klagenfurt am Wörthersee, Austria; 8 3rd Medical Department, Hematology and Oncology, Hanusch Hospital, Heinrich-Collin-Straße 30, 1140 Vienna, Austria; 9 Department of Gynecology, Breast Cancer Center Vienna, Medical University of Vienna, Währinger Gürtel 18–20, 1090 Vienna, Austria

**Keywords:** HER2, Trastuzumab, Pertuzumab, Antibody-conjugate, Tyrosin-kinase inhibitor

## Abstract

A group of Austrian breast cancer specialists met in December 2020 to establish a comprehensive clinical benefit-risk profile of available HER2-targeted therapies based on recent data and to develop an updated treatment algorithm by consensus over several months in 2021. A total of four scenarios were developed in which treatment strategies appropriate for specific patient profiles were evaluated. Consensus was established by detailed discussions of each scenario and by reaching full consensus.

**Supplementary Information:**

The online version of this article (10.1007/s00508-021-01987-9) contains supplementary material, which is available to authorized users.

## Introduction

HER2-positive breast cancer, characterized by the overexpression of the HER2 protein and/or amplification of the* HER2/neu *gene has experienced a significant growth in treatment options over the last two decades. HER2, a ligandless growth factor receptor of the EGFR family, was established as a negative prognostic marker in breast cancer in the 1980s [1]. The introduction of the monoclonal antibody trastuzumab as the first HER2-specific targeted therapy led to tremendous improvement in recurrence-free survival and overall survival (OS) in early breast cancer [2] and progression-free survival (PFS) and OS in metastatic disease [3]. Pertuzumab [4, 5], another monoclonal antibody, and the antibody-drug conjugate (ADC) trastuzumab emtansine (T-DM1) [6–9] have led to a further improvement in outcomes. In addition, the tyrosine kinase inhibitors (TKIs) lapatinib and neratinib are also available for the treatment of HER2-positive breast cancer (Fig. 6; [10–13]).

Today, trastuzumab, pertuzumab, and T‑DM1 [9] are routinely used in the (neo)adjuvant and postneoadjuvant settings, which may result in limited treatment options in cases of disease recurrence; additional options are also required in patients progressing on standard therapy. With the recent approvals of trastuzumab-deruxtecan [14, 15] and tucatinib [16, 17] (as a triple combination with capecitabine and trastuzumab), two novel drugs have become available: on 18 January 2021, the European Commission granted conditional approval for the ADC trastuzumab-deruxtecan [15]. For the TKI tucatinib, conditional approval was granted on 12 February 2021 [17]. Both compounds have been evaluated in several clinical trials in recent years [18–25], necessitating the development of revised treatment recommendations.

## Patients, material and methods

A group of Austrian breast cancer specialists met in December 2020 to establish a comprehensive clinical benefit-risk profile of available HER2-targeted therapies based on recent data and to develop an updated treatment algorithm by consensus over several months in 2021.

The basis of the scientific-clinical review of the therapeutic options were data from the following sources: all studies included in the consensus statement, regulatory information on established and new compounds, scientific updates of the last years from the following symposia/congresses: San Antonio Breast Cancer Symposium, the American Society of Clinical Oncology Annual Meetings, the European Society for Medical Oncology Annual Meetings, safety profiles and efficacy data of the respective compounds, current treatment recommendations for patients with metastatic HER2+ breast cancer from various guidelines, and comprehensive clinical practice experiences of the respective experts, their teams and institutions.

**Fig. 1:**
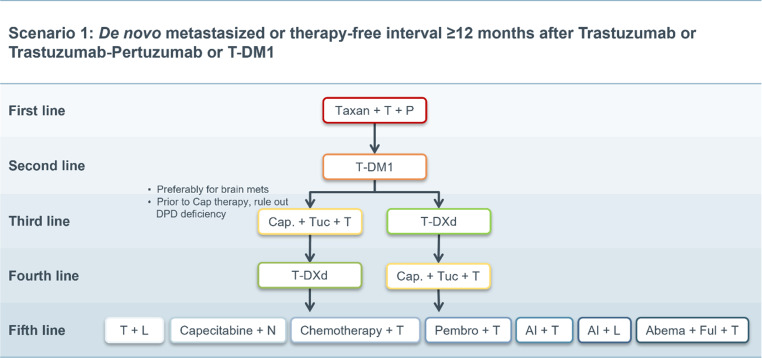
Scenario 1. *T* trastuzumab, *P* pertuzumab, *T‑DM1* trastuzumab emtansine, *Cap* capecitabine, *Tuc* tucatinib, *T‑DXd* trastuzumab deruxtecan, *L* lapatinib, *N* neratinib, *Pembro* pembrolizumab, *AI* aromatase inhibitor, *Abema* abemaciclib, *Ful* fulvestrant, *brain mets* brain metastases, *DPD* Dihydropyrimidin-Dehydrogenase

**Fig. 2:**
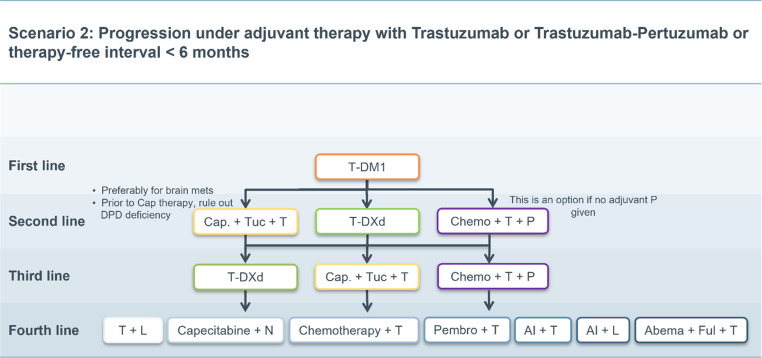
Scenario 2. *T‑DM1* trastuzumab emtansine, *Cap* capecitabine, *Tuc* tucatinib, *T‑DXd* trastuzumab deruxtecan, *Chemo* chemotherapy, *T* trastuzumab, *P* pertuzumab *L* lapatinib, *N* neratinib, *Pembro* pembrolizumab, *AI* aromatase inhibitor, *Abema* abemaciclib, *Ful* fulvestrant, *brain mets* brain metastases, *DPD* Dihydropyrimidin-Dehydrogenase

**Fig. 3:**
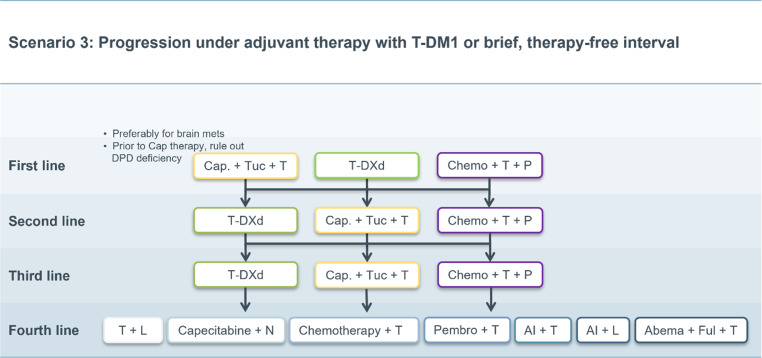
Scenario 3. *Cap* capecitabine, *Tuc* tucatinib, *T‑DXd* trastuzumab-deruxtecan, *Chemo* chemotherapy, *AI* aromatase inhibitor, *T* trastuzumab, *P* pertuzumab *L* lapatinib, *N* neratinib, *Pembro* pembrolizumab, *Abema* abemaciclib, *Ful* fulvestrant, *brain mets* brain metastases, *DPD* Dihydropyrimidin-Dehydrogenase

**Fig. 4:**
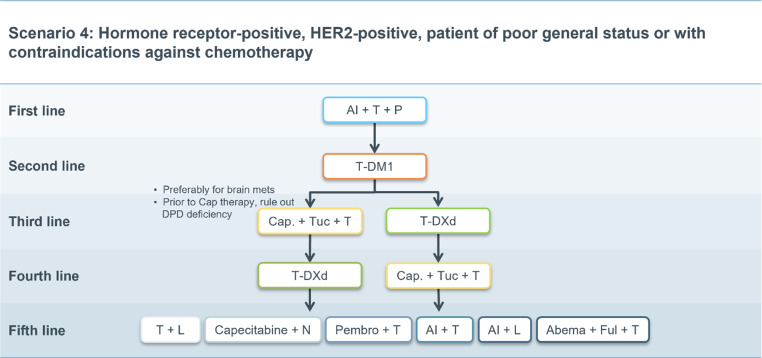
Scenario 4. *AI* aromatase inhibitor, *T* trastuzumab, *P* pertuzumab, *T‑DM1* trastuzumab emtansine, *Cap* capecitabine, *Tuc* tucatinib, *T‑DXd* trastuzumab deruxtecan, *L* lapatinib, *N* neratinib, *Pembro* pembrolizumab, *Abema* abemaciclib, *Ful* fulvestrant, *brain mets* brain metastases, *DPD* Dihydropyrimidin-Dehydrogenase

A total of four scenarios were developed in which treatment strategies appropriate for specific patient profiles were evaluated. Consensus was established by detailed discussions of each scenario and by reaching full consensus (see Figs. 1, 2, 3, and 4).

## Results

### Active substances, modes of action and clinical trial landscape

Sequential administration of the currently available therapeutic agents has dramatically increased survival in patients with HER2-positive metastatic breast cancer [26] but as previously discussed, novel drugs are still required. Improved understanding of HER2 signaling, immunomodulatory mechanisms, ADC technology, and basic tumor biology has enabled the development of next-generation HER2-targeted compounds.

**Fig. 5:**
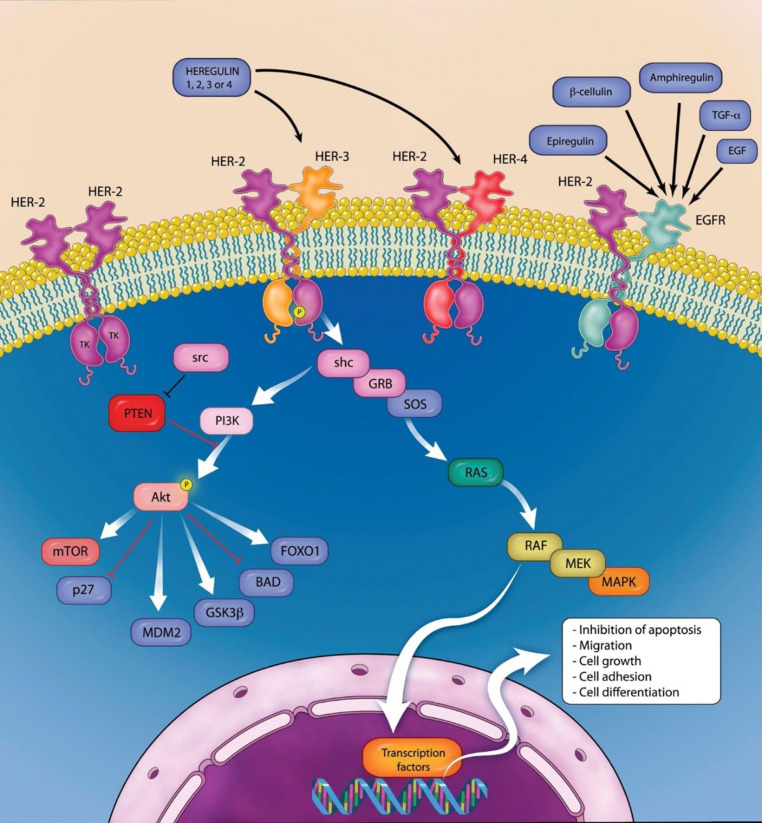
Simplified signal cascade of HER family growth factor receptors [29]. *Akt* RAC-alpha serine/threonine-protein kinase, *BAD* Bcl2-associated agonist of cell death, *EGFR* epidermal growth factor receptor, *FOXO1* forkhead box protein O1, *GRB* growth factor receptor bound protein, *GSK3ß* glycogen synthase kinase 3 beta, *HER2* human epidermal growth factor receptor 2, *MAPK* mitogen-activated protein kinase, *MDM2* mouse double minute 2 oncogene, *MEK* MAP extracellular kinase, *mTOR* Mammalian target of rapamycin, p27 Cyclin-dependent kinase inhibitor 1B, *PI3K* phosphoinositide 3‑kinase, *PTEN* phosphatase tensin homolog, *RAF* rapidly accelerated fibrosarcoma protooncogene, *RAS* rat sarcoma oncogene, *SHC* Src homog adaptor protein, *SOS* son of sevenless homolog/guanine nucleotide exchange factor, *src* sarcoma protooncogene, *TGF‑α* transforming growth factor alpha (courtesy of Sage Publishing and Alessandro Baliani, Copyright © 2012. Original publication: Okines AF, Cunningham D. Trastuzumab: a novel standard option for patients with HER-2-positive advanced gastric or gastroesophageal junction cancer. Therap Adv Gastroenterol. 2012 Sep;5(5):301–318. PMID: 22973416)

HER2 belongs to the transmembrane tyrosine kinase receptors and inhibits apoptosis via the mTOR pathway and stimulates cell proliferation via the RAS-MAPK. pathway [27, 28]. An overview of HER2 homodimers and heterodimers and the associated signaling cascades is shown in Fig. 5.

**Fig. 6:**
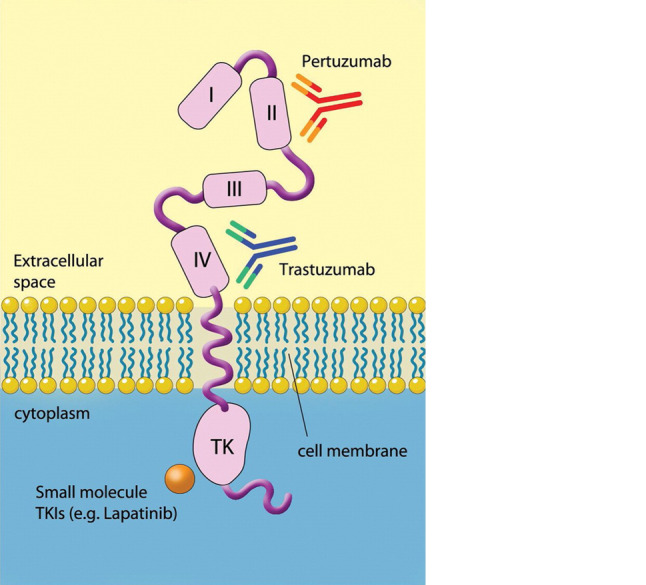
HER2 dual inhibition by trastuzumab and pertuzumab and intracellular targeting of the HER2 tyrosine kinase domain by HER2-directed TKIs [29]. ADCs, such as T‑DXd and T‑DM1, target the extracellular domain and exert their additional activity after internalization of the antibody/antigen complex and liberation of the toxic *payload. TKIs* tyrosine kinase inhibitors (lapatinib, neratinib, tucatinib), *ADC* antibody-drug conjugate, *T‑DXd* trastuzumab deruxtecan, *T‑DM1* trastuzumab emtansine (courtesy of Sage Publishing and Alessandro Baliani, Copyright © 2012. Original publication: Okines AF, Cunningham D. Trastuzumab: a novel standard option for patients with HER-2-positive advanced gastric or gastroesophageal junction cancer. Therap Adv Gastroenterol. 2012 Sep;5(5):301–18. PMID: 22973416)

The clinical consequence of HER2 protein overexpression and/or *HER2/neu *amplification is a biologically aggressive phenotype; 15–20% of primary breast cancers are HER2-positive [1, 3, 30].

Supplementary Table 1 summarizes HER2-targeted agents, their respective mechanisms of action, regulatory information, and treatment indications based on publicly available information from the European Medicines Agency and recent studies.

Supplementary Table 2 provides an overview of the most important studies in HER2+ breast cancer that form the basis for the current standard of care.

### Development of updated treatment algorithms and consensus scenarios

The scenarios and respective treatment algorithms depicted here were developed by consensus and are based on all available data in the scientific literature about HER2-positive breast cancer. In early treatment lines, most treatment recommendations were delineated by the design and inclusion criteria of the respective clinical trials. An exception to this arises from the inclusion criteria of the CLEOPATRA [4, 5] and EMILIA [6, 8] trials. Since CLEOPATRA only included patients with a therapy-free interval of at least 12 months after the end of adjuvant antibody therapy and EMILIA included first-line patients with a therapy-free interval of up to 6 months, there is an artificial evidence gap in the interval of > 6 and < 12 months. In cases of recurrence within this interval, there is insufficient data to provide general treatment recommendations. Decisions in such cases must be made individually depending on factors such as response to neoadjuvant therapy, prior therapeutic regimens, timing of recurrence, and patient-specific factors. In general, a biopsy for re-evaluation of tumor biology should be sought in any recurrency scenario regardless of the time interval between surgery and diagnosis of metastatic disease.

### Scenario 1

In de novo metastatic patients or patients with a treatment-free interval of ≥ 12 months after the end of adjuvant therapy, all available agents are available according to current guidelines [31, 32].

First-line therapy is provided according to data generated by the CLEOPATRA study with trastuzumab plus pertuzumab plus taxane-based chemotherapy (e.g., docetaxel). Dual HER2-inhibition plus chemotherapy was shown to generate a clinically relevant OS advantage over the former treatment standard of trastuzumab plus docetaxel by a median of 16.3 months (HR: 0.69; 95% CI; 0.58–0.82) [5]. For hormone receptor (HR)-positive, HER2-positive tumors (luminal B/HER2-positive; triple positive), based on the ESMO/ABC guidelines, the addition of endocrine therapy to antibody maintenance therapy after completion of induction chemotherapy is recommended. Chemotherapy-free treatment consisting of endocrine therapy plus HER2-directed therapy is not considered a standard first-line approach and should be reserved for selected patients and not deemed a candidate for standard therapy (*see also* scenario 4).

In the second-line setting T‑DM1 is considered as the standard of care based upon data from the EMILIA [6] and TH3RESA [7] trials. Compared with the already outdated former second-line standard capecitabine plus lapatinib, T‑DM1 showed a significant advantage in median OS of 4 months (HR: 0.75; 95% CI; 0.64–0.88) despite cross-over with a favorable toxicity profile [6]. It should be noted that a smaller absolute benefit of T‑DM1 can be assumed after pretreatment with trastuzumab and pertuzumab [33].

In this scenario, T‑DXd or tucatinib (as a triple combination therapy with trastuzumab and capecitabine) can both be applied as third-line treatment based upon results from the DESTINY-Breast01 and HER2climb trials [15, 17].

The DESTINY-Breast01 study, a single-arm phase II trial, evaluated the efficacy and safety of T‑DXd in a heavily pretreated population with median of 6 prior therapy lines in the metastatic setting. Single-agent T‑DXd yielded an objective response rate of 61.4%, median progression-free survival was 19.4 months, and median OS was estimated at 24.6 months with a median follow-up of 20.5 months. All patients had received prior therapy with T‑DM1 indicating that T‑DXd can overcome ADC resistance. In the subgroup of 24 patients with stable CNS metastases at study inclusion (17 with brain lesions at baseline), extracranial objective response rate was 58.3%, and median progression-free survival 18.1 months; data regarding a potential intracranial activity of T‑DXd in brain metastases are insufficient at this time. The most common adverse events with T‑DXd monotherapy were nausea (77%), fatigue (49.5%), alopecia (48.4%), and vomiting (45.7%). Pulmonary adverse events occurred in the form of interstitial lung disease (ILD)/pneumonitis in 15.2% (3.2% grade 3 or higher) of patients and resulted in death in a total of 5 study participants. In this respect, appropriate monitoring and patient education as well as rapid therapeutic intervention with corticosteroids are required when ILD is suspected [20, 22]. T‑DXd is currently being further evaluated in a broad study program [34].

In the randomized phase II HER2climb study, the triple combination of trastuzumab plus capecitabine plus tucatinib showed a significant benefit in median progression-free survival of 2.2 months compared with trastuzumab, capecitabine, and placebo (HR: 0.54; 95% CI; 0.42–0.71; *P* < 0.001) in a heavily pretreated population who had all progressed on prior treatment with trastuzumab. pertuzumab and T‑DM1. Median OS was prolonged by 4.5 months (HR: 0.66; 95% CI; *P* = 0.005). Of particular note, 47.5% of patients in HER2climb had brain metastases at baseline, of which 59.8% were considered active (i.e., newly diagnosed or progressive after prior local therapy). These subgroups also benefited from the addition of tucatinib (OS for active brain metastasis: HR 0.49; 95% CI; 0.30–0.80). This third generation TKI has a significantly lower rate of severe diarrhea compared with older agents due to its high specificity to the tyrosine kinase domain of HER2; still, diarrhea occurred in 80.9% of patients but diarrhea ≥ grade 3 was relatively rare at 12.9%. Transaminase elevations were more common in the tucatinib arm but were mostly low-grade, transient, and reversible [17, 18, 25].

A direct comparison of the two therapeutic options is not available at the time of publication. In this respect, the weighing and assessment of benefits and risks must be made based on available data. Both substances yielded high relevant clinical activity, and tucatinib is the first drug to generate a significant OS improvement over chemotherapy plus trastuzumab in a heavily pretreated population; in addition, intriguing activity was observed in patients with active brain metastases. Response rates and duration of response of T‑DXd shown in DESTINY-Breast01 are currently unprecedented in a heavily pretreated patient population; however, it should be noted that DESTINY-Breast01 is a single-arm phase II study and data from the randomized DESTINY-Breast02 study are still pending.

For patients with intracranial metastases, the triple combination with trastuzumab plus capecitabine plus tucatinib is currently preferred, as the data for T‑DXd are insufficient in this context. For patients with pre-existing severe pulmonary disease, an appropriate risk assessment is recommended when considering the use of T‑DXd. Patient preference and compliance aspects with different treatment modalities (i.v. vs. oral; treatment once every 3 weeks vs. daily drug intake) must also be considered in the clinical decision making.

Upon progression on T‑DXd or tucatinib the other of the two newly approved substances not utilized in the third-line setting should be considered. At the time of publication, no data were available on the optimal sequencing of these two options.

Beyond the fourth line, no clear therapeutic recommendation according to the current guidelines [31, 32, 35] and the expert assessments can be given. Therefore, the individual needs of the patients as well as disease-specific factors must be considered in order to achieve the best possible outcome. Data from numerous studies suggest that in any case, continuation of HER2-targeted therapy is beneficial [36–39].

Lapatinib in combination with trastuzumab (vertical dual blockade) or capecitabine is a standard option in the treatment of HER2-positive metastatic breast cancer [6, 10, 11, 40, 41]; however, the NALA study showed that neratinib plus capecitabine was significantly superior to lapatinib plus capecitabine in terms of PFS (HR: 0.76; 95% CI; 0.63–0.93; *P* = 0.0059) [13]. In patients with brain metastases, both TKIs are considered established treatment options [13]. On the downside, both drugs are characterized by high diarrhea rates. In addition, neratinib is currently approved only by the FDA for the use in metastatic breast cancer patients. In addition, no data on therapy with lapatinib or neratinib after tucatinib are currently available. Thus, the accepted concept of treatment with trastuzumab in multiple lines with alternating chemotherapeutic combination partners appears to represent a potential standard from the fifth treatment line onwards.

In the case of luminal B/HER2-positive disease, additional treatment options may be considered: In the phase II monarcHER trial, the combination of abemaciclib, fulvestrant and trastuzumab was shown to prolong PFS by 2.6 months compared with trastuzumab and chemotherapy (HR: 0.67; 95% CI; *P* = 0.051) [42]. Currently however, there is no approval for abemaciclib in HER2-positive disease. A combination of endocrine therapy and trastuzumab is also possible, but data supporting this therapeutic approach in later lines of therapy are limited.

The combination of the PD‑1 inhibitor pembrolizumab with trastuzumab is another individual option lacking formal approval. In the single-arm phase 1b-2 study PANACEA, the efficacy and safety of the antibody combination was investigated in patients with trastuzumab resistance. Treatment was generally well-tolerated, and 15% of PD-L1 positive patients showed an objective response (90% CI: 7–29%) with higher response rates in the PD-L1/tumor-infiltrating lymphocyte co-positive subgroup [43]. Fig. 1 shows an adapted therapy algorithm based on the most recent data and current approvals in the therapy of metastatic breast cancer.

### Scenario 2

In patients with progression under adjuvant therapy with trastuzumab or trastuzumab plus pertuzumab or relapse within 6 months after the end of adjuvant therapy, a rechallenge with trastuzumab, pertuzumab and chemotherapy in the first-line setting does not seem reasonable. Therefore, T‑DM1 is recommended as first-line therapy [6].

Subsequently, all other therapy options described in the standard scenario above move up by one therapy line, thus, according to recent market authorizations, there is the possibility of using the two new substances T‑DXd or tucatinib already in the second-line setting, as shown in Fig. 2 [18, 20, 22].

Alternatively, a rechallenge with trastuzumab plus pertuzumab in combination with chemotherapy in the 2nd, 3rd or 4th line can be considered. This seems particularly relevant in patients who have not yet received adjuvant pertuzumab or responded favorably to neoadjuvant treatment.

The same considerations as in scenario 1 apply to further therapy lines.

### Scenario 3

A special situation arises in cases of systemic relapse during post neoadjuvant therapy with T‑DM1 or relapse within a treatment-free interval of 6 months or less.

If these patients had already received trastuzumab and pertuzumab in the neoadjuvant setting, the benefit of rechallenging with trastuzumab, pertuzumab and chemotherapy seems questionable. This may be assessed differently if a patient with an initially large disease burden did not achieve pathological complete remission despite having favorable treatment response.

According to market authorization language, in this scenario there is the option to use the two newly approved compounds already in the first or second line (Fig. 3).

After second-line therapy (or third-line therapy if a rechallenge with trastuzumab + pertuzumab + chemotherapy was deemed appropriate), all therapeutic options described in the standard scenario subsequently move up by one line.

### Scenario 4

Patients who have contraindications to chemotherapy represent a specific scenario. In this case, the therapeutic algorithm may be adapted accordingly as shown in Fig. 4.

In patients with luminal B/HER2-positive breast cancer, a combination of trastuzumab, pertuzumab and an aromatase inhibitor (AI) can be used as first-line therapy based upon the PERTAIN study [44]. Results from the ALTERNATIVE trial have shown that in this population, PFS is also significantly prolonged with lapatinib, trastuzumab and an AI [41] over single agent HER2 inhibition with either trastuzumab or lapatinib plus endocrine therapy. Data from the phase II VELVET trial have shown that first-line combination therapy with trastuzumab, pertuzumab, and vinorelbine is a safe alternative to standard (taxane-based) chemotherapy [45, 46]. More recently, a randomized trial demonstrated that in old and frail patients, the addition of metronomic oral cyclophosphamide to trastuzumab and pertuzumab can prolong median progression-free survival by 7 months compared with antibody therapy alone [47]. In the second-line setting, T‑DM1 is also the standard of care in this patient population due to its acceptable toxicity profile [6]. Thereafter, T‑DXd or tucatinib [18, 20, 22, 48] may both be considered, although specific data on the use of third-generation agents in an old and/or comorbid population are still pending.

## Discussion and outlook

The broad use of trastuzumab, pertuzumab, and T‑DM1 [9] in the adjuvant and neoadjuvant settings increasingly creates situations where treatment options are limited in patients with metachronous metastatic disease. In addition, new treatment options are required in the metastatic setting in patients progressing on current standard therapies. With trastuzumab-deruxtecan [15] and tucatinib [17] two novel, highly effective agents have become available. T‑DXd and tucatinib both received conditional approval in early 2021 and have been studied in several clinical trials in recent years [18, 21–23, 25, 48]. Data show that both agents have compelling activity in pretreated patients. Regarding T‑DXd, response rates and progression-free survival are unprecedented in a heavily pretreated patient population but results are derived from a single-arm phase II study and data from randomized trials are pending. Tucatinib is characterized by a clinically relevant prolongation of PFS and OS in a randomized phase 2 study in patients progressing on all current standard treatment options, and major activity was also observed in patients with active brain metastases [18, 25]. Currently, both compounds are approved after two prior lines of HER2-directed treatment, including (neo)adjuvant therapy. Therefore, these agents have defined a novel treatment standard from the third-line setting but may be used in earlier treatment lines depending upon individual pretreatment and course of disease. Numerous studies are currently ongoing and future data on the optimal use and treatment sequence of T‑DXd and tucatinib are expected soon.

## Supplementary Information

**Supplementary Table 1: **Description of selected HER2+ breast cancer agents grouped by antibody, antibody-drug conjugates (ADC) and tyrosine kinase inhibitors (TKIs).

**Supplementary Table 2: **Overview of selected studies in the neo-/adjuvant or metastatic setting.

## Abbreviations

Abe/Abema: Abemaciclib
Act: RAC-alpha serine/threonine-protein kinase
ADC: Antibody-drug conjugate
ADCC: Antibody-dependent cell-mediated cytotoxicity
Adj: Adjuvant
AE/SAE: Adverse event/serious adverse event
AI: Aromatase inhibitor
AK: Antibody
Ana: Anastrozole
BAD: Bcl2-associated agonist of cell death
Cap: Capecitabine
Carbo: Carboplatin
CBR: Clinical benefit ratio
CHMP: Committee for Medicinal Products for Human Use of the EMA
ChT/Chemo: Chemotherapy
CI: Confidence interval
CNS: Central nervous system
Cyclo: Cyclophosphamide
DFS: Disease-free survival
Doce: Docetaxel
Doxo: Doxorubicin
DPD: Dihydropyrimidine dehydrogenase
EGFR: Epidermal growth factor receptor
EMA: European Medicines Agency
FDA: U.S. Food and Drug Administration
FEC: Fluorouracil, epirubicin, cyclophosphamide
FOXO1: Forkhead box protein O1
Ful: Fulvestrant
GRB: Growth factor receptor-binding protein
GSK3ß: Glycogen synthase kinase 3 beta
HER2: Human epidermal growth factor receptor 2
HFS: Hand-foot syndrome
HR: Hazard ratio
HRec: Hormone receptor
iDFS: Invasive disease-free survival
ILD: Interstitial lung disease
IQR: Interquartile range
L/Lap: Lapatinib
Letro: Letrozole
LVEF: Left ventricular ejection fraction
MAPK: Mitogen-activated protein kinase
MCC: 4-[N-maleimidomethyl]cyclohexane-1-carboxylate
MDM2: Mouse double minute 2 Oncogene
MEK: MAP Extracellular kinase
mTOR: Mammalian target of rapamycin
N/Ner: Neratinib
Neo/Neo-adj: Neoadjuvant
NYHA: New York Heart Association
ORR: Objective response rate
OS: Overall survival
*P*: Pertuzumab
p27: Cyclin-dependent kinase inhibitor 1B
pCR: Pathological complete response
Pem/Pembro: Pembrolizumab
PFS: Progression-free survival
PI3K: Phosphoinositide 3‑kinase
PTEN: Phosphatase tensin homolog
RAF: Rapidly accelerated fibrosarcoma protooncogene
RAS: Council sarcoma oncogene
SHC: Src homog adaptor protein
SOC: Standard of care/treatment standard of the center
SOS: Son of sevenless homolog/Guanine nucleotide exchange factor
Src: Sarcoma protooncogene
T: Trastuzumab
TBP: Trastuzumab beyond progression
T‑DM1: Trastuzumab emtansine
T‑DXd: Trastuzumab-deruxtecan
TGF‑α: Transforming growth factor alpha
TKI: Tyrosine kinase inhibitor
TTP: Time to progression
Tuc/TUC: Tucatinib
