# Supplementary material for: Updated Austrian treatment algorithm in HER2+ metastatic breast cancer
Source: Wien Klin Wochenschr. 2022 Jan 28;134(1-2):63–72. doi: 10.1007/s00508-021-01987-9 (PMC8813714; doi:10.1007/s00508-021-01987-9)
Supplement: Supplementary file 1 — Supplementary Table 1: Description of selected HER2+ breast cancer agents grouped by antibody, antibody-drug conjugates (ADC) and tyrosine kinase inhibitors (TKIs). [file 508_2021_1987_MOESM1_ESM.docx]

| **ANTIBODY** | |
| --- | --- |
| **Trastuzumab (2, 29, 37-39, 49, 50)** | |
| **Mechanism of action**  Trastuzumab is a recombinant humanized IgG1 monoclonal antibody against HER2.  Trastuzumab binds with high affinity and specificity to HER2 subdomain IV, a juxtamembrane region in the extracellular domain of HER2. Binding of trastuzumab to HER2 inhibits ligand-independent HER2 signaling and prevents proteolytic cleavage of the extracellular domain, an activation mechanism of HER2. Accordingly, trastuzumab has been shown to inhibit proliferation of human tumor cells overexpressing HER2 in both in vitro experiments and animal models. In addition, trastuzumab is a potent mediator of antibody-dependent cell-mediated cytotoxicity (ADCC). *In vitro,* trastuzumab-mediated ADCC has been shown to act preferentially on HER2-overexpressing cancer cells compared to cancer cells without HER2 overexpression. | **Approval status and application**  Approval number(s) EU/1/00/145/002  Date of issue of approval: August 28, 2000  Date of last renewal of approval: August 28, 2010  Trastuzumab is indicated for the treatment of adult patients with HER2-positive metastatic breast cancer:   - As monotherapy after at least two chemotherapy regimens and hormone treatment in hormone receptor-positive patients. - In combination with paclitaxel without prior chemotherapy in the metastatic setting. - In combination with docetaxel without prior chemotherapy in the metastatic setting. - in combination with an aromatase inhibitor for the treatment of postmenopausal patients with hormone receptor-positive metastatic breast cancer who have not yet been treated with trastuzumab.   HER2-positive early breast cancer:   - After surgery, chemotherapy (neoadjuvant or adjuvant) and radiotherapy (if applicable). - After adjuvant chemotherapy with doxorubicin and cyclophosphamide, in combination with paclitaxel or docetaxel. - in combination with adjuvant chemotherapy with docetaxel and carboplatin - In combination with neoadjuvant chemotherapy followed by adjuvant therapy with trastuzumab, for locally advanced (including inflammatory) breast cancer or tumors >2 cm in diameter. |
|  | |
| **Pertuzumab (4, 5, 51-53)** | |
| **Mechanism of action**  Pertuzumab is a recombinant humanized monoclonal antibody that specifically binds to extracellular subdomain II of HER2, inhibiting ligand-dependent heterodimerization of HER2 with other HER receptor family receptors, including EGFR, HER3 and HER4. Thus, pertuzumab inhibits ligand-dependent intracellular signaling through two major signaling pathways, that of mitogen-activated protein kinase (MAP) and that of phosphoinositide 3-kinase (PI3K). Similarly, signaling is inhibited in a ligand-independent manner as HER2 is constitutionally activated (54). Inhibition of each of these signaling pathways can lead to cellular growth arrest or apoptosis. In addition, pertuzumab is a mediator of antibody-dependent cell-mediated cytotoxicity (ADCC). While pertuzumab alone inhibited human tumor cell proliferation, the combination of pertuzumab and trastuzumab significantly increased antitumor activity in HER2-overexpressing heterograft models. | **Approval status and application**  Approval number(s): EU/1/13/813/001  Date of first granting of approval: 04 March 2013  Date of the last renewal of the approval: 08 December 2017  HER2-positive early breast cancer:  Pertuzumab is indicated for use in combination with trastuzumab and chemotherapy for:   - neoadjuvant treatment of adult patients with HER2-positive locally advanced, inflammatory or early breast cancer at high risk of recurrence. - adjuvant treatment of adult patients with HER2-positive early breast cancer at high risk of recurrence.   Metastatic breast cancer  Pertuzumab is indicated for use in combination with trastuzumab and docetaxel in adult patients with HER2-positive metastatic or locally recurrent unresectable breast cancer who have not previously received anti-HER2 therapy or chemotherapy for the treatment of their metastatic disease. |
|  | |
| **ANTIBODY-DRUG CONJUGATES (ADC)** | |
| **Trastuzumab emtansine (T-DM1) (6-9, 55)** | |
| **Mechanism of action**  T-DM1, is an anti-HER2 ADC and contains trastuzumab, a humanized anti-HER2 AK covalently linked to the microtubule inhibitor DM1 (referred to as *payload*) via the stable thioether linker MCC (4-[N-maleimidomethyl]cyclohexane-1-carboxylate). On average, 3.5 DM1 molecules are bound to each molecule of trastuzumab (*drug-to-antibody* ratio 3.5:1). Conjugation of DM1 to trastuzumab causes selectivity for tumor cells overexpressing HER2. After binding to HER2, T-DM1 undergoes receptor-mediated internalization and subsequent lysosomal degradation, resulting in the release of DM1-containing cytotoxic catabolites.  T-DM1 has both the mechanism of action of trastuzumab and DM1.  DM1, the cytotoxic component of T-DM1, binds to tubulin. By inhibiting the polymerization of tubulin, both DM1 and T-DM1 cause cells to arrest in the G2/M phase of the cell cycle, ultimately leading to apoptosis. | **Approval status and application**  Approval number(s): EU/1/13/885/001,  EU/1/13/885/002  Date of issue of approval: November 15, 2013  Date of the last renewal of the authorization: September 17, 2018  HER2-positive early breast cancer:  T-DM1 is used as a single agent for adjuvant treatment in adult patients with HER2-positive early breast cancer who have invasive residual disease in the breast and/or lymph nodes following neoadjuvant taxane-based and HER2-targeted therapy.  Metastatic breast cancer  T-DM1 is used as a single agent for the treatment of adult patients with HER2+, unresectable locally advanced or metastatic breast cancer who have previously, individually  or in combination, have received trastuzumab and a taxane. Patients should either:   - Have received prior treatment for locally advanced or metastatic disease; or - Developed a recurrence during or within six months of completion of adjuvant treatment. |
|  | |
| **Trastuzumab-deruxtecan (T-DXd) (19-22, 56, 57)** | |
| **Mechanism of action**  Trastuzumab-deruxtecan is a monoclonal ADC directed against HER2. T-DXd contains trastuzumab, a humanized anti-HER2 AK. The potent topoisomerase I inhibitor (payload) is bound to trastuzumab via a cleavable linker with a high *drug-to-antibody* ratio (8:1). Deruxtecan has a high membrane permeability, thus exhibiting a cytotoxic effect against neighboring cells (*bystander effect*). After internalization, the released DXd induces tumor cell apoptosis. | **Approval status and application**  Approval number(s): EU/1/20/1508(001)  Date of granting of approval: January 18, 2021  Trastuzumab-deruxtecan is used as monotherapy for the treatment of adult patients with unresectable or metastatic HER2-positive breast cancer who have already received at least two treatments for HER2-positive breast cancer. |
|  | |
| **TYROSINE KINASE INHIBITORS (TKI)** | |
| **Lapatinib (6, 8, 10, 13, 35, 39, 58, 59)** | |
| **Mechanism of action**  Lapatinib, a 4-anilinoquinazoline, is an inhibitor of the intracellular tyrosine kinase domains, ErbB1 and HER2.  Lapatinib inhibits ERBB-dependent tumor cell growth. The combination of lapatinib and trastuzumab provides complementary mechanisms of action as well as potentially non-overlapping resistance mechanisms. The growth inhibitory effect of lapatinib was tested in cell lines pretreated with trastuzumab. Lapatinib retained a significant activity against breast cancer cell lines with HER2 amplification selected for long-term growth in *vitro in* a trastuzumab-containing medium and acted synergistically in combination with trastuzumab in these cell lines. | **Approval status and application**  Approval number(s): EU/1/07/440/001-007  Date of issue of approval: June 10, 2008  Date of last renewal of approval: February 17, 2015  Lapatinib is indicated for the treatment of adult patients with breast cancer whose tumors overexpress HER2;   - In combination with capecitabine in patients with advanced or metastatic disease that is progressive after prior therapy that included anthracyclines and taxanes and, in the metastatic setting, trastuzumab. - In combination with trastuzumab in patients with hormone receptor-negative metastatic disease that is progressive after prior trastuzumab therapy(s) in combination with chemotherapy. - In combination with an aromatase inhibitor in postmenopausal women with hormone receptor-positive metastatic disease not currently scheduled for chemotherapy. |
|  | |
| **Neratinib (12, 13, 60)** | |
| **Mechanism of action**  Neratinib is an irreversible pan-ERBB TKI that inhibits mitogenic growth factor signal transduction by covalent (irreversible) binding with high affinity at the ATP-binding site of (among others) EGFR, HER2 and HER4, thereby inhibiting downstream MAPK and AKT signaling pathways. In addition to HER2 overexpressing tumors, neratinib exhibits activity against specific HER2 mutations. | **Approval status and application**  Approval number(s): EU/1/18/1311/001  Date of issue of approval: August 31, 2018  Neratinib is indicated for the extended adjuvant treatment of adult patients with hormone receptor-positive, HER2-overexpressed/amplified early-stage breast cancer who completed trastuzumab-based adjuvant therapy less than one year ago. |
|  | |
| **Tucatinib (16, 17, 25, 61)** | |
| **Mechanism of action**  Tucatinib is an irreversible TKI that blocks the tyrosine kinase domain of HER2 with high specificity and has significantly lower inhibitory activity against EGFR resulting in a reduced diarrhea rate compared to lapatinib and neratinib. Tucatinib inhibits HER2 signaling and consequently cell proliferation and induces tumor cell apoptosis. | **Approval status and application**  Approval number(s):  50 mg film-coated tablets: EU/1/20/1526/001  150 mg film-coated tablets: EU/1/20/1526/002  On February 12, 2021, the European Commission conditionally approved tucatinib for the treatment of HER2+, locally advanced or metastatic breast cancer. At the time of publication, the information was not yet available on the European Commission site.  Tucatinib is indicated in combination with trastuzumab and capecitabine in the treatment of adult patients with HER2+, locally advanced or metastatic breast cancer:   - In patients who have had at least 2 prior anti-HER2 treatments. |

**Supplementary Table 1:** Description of selected HER2+ breast cancer agents grouped by antibody, antibody-drug conjugates (ADC) and tyrosine kinase inhibitors (TKIs).
