# Supplementary material for: Updated Austrian treatment algorithm in HER2+ metastatic breast cancer
Source: Wien Klin Wochenschr. 2022 Jan 28;134(1-2):63–72. doi: 10.1007/s00508-021-01987-9 (PMC8813714; doi:10.1007/s00508-021-01987-9)
Supplement: Supplementary file 2 — Supplementary Table 2: Overview of selected studies in the neo-/adjuvant or metastatic setting. [file 508_2021_1987_MOESM2_ESM.docx]

| **Studies in the adjuvant setting** | | | | |
| --- | --- | --- | --- | --- |
| **Acronym**  **Author, Year, Journal** | **Substances** | **Patients**  **Design** | **Results** | **Comments** |
| **HERA (2, 62)**  Piccart-Gebhart et al, 2005, NEJM. | T (1 or) 2 years vs. control after adj ChT | 1694 patients with T vs. 1693 patients under observation  Open, phase III, prospective, randomized, multicenter | Primary endpoint: PFS  Significant improvement in PFS; HR 0.54 (95% CI, 0.43-0.67; P<0.0001);  2-year DFS significantly better in T group at 8.4%; OS not significantly better (29 vs. 37 deaths favoring T group); Severe cardiotoxicity observed in 0.5% of T group patients. No additional benefit under 2-year T therapy vs. 1 year T.  OS benefit in 11-year analysis, 73% in observation group, 79% in T group (1Y), and 80% in 2-year T group (HR 0.74 (95% CI; 0.64 - 0.86). | Adjuvant chemotherapy with or without trastuzumab (additional randomization to trastuzumab for 1 year or 2 years). |
| **NSABP-B31 (63)**  Romond et al, 2005, NEJM. | Gr1: Doxo+Cyclo, then Paclitaxel.  Gr2: Gr1 + T with Paclitaxel | Total 3351 patients  B31 Study:  T Arm: 864 Pat  Control: 872 Pat  N9831 Study:  T Arm: 808 Pat  Control: 807 Pat  Open, phase III, prospective, randomized, multicenter | Primary endpoint: DFS  2-year follow-up (2.4 for B31; 1.5 for N9831): 261 events in control vs 133 in T arm; (HR: 0.48 ;95% CI; 0.39-0.59; P <0.0001).  3-year DFS: 75.4% in the control arm vs. 87.1% in T (95% CI).  4-year DFS: 67.1% in the control arm vs 85.3% in T (HR: 0.48; 95% CI; P<0.001).  OS at 4 years: 86.6% vs 91.4% in favor of T (HR: 0.67; 95% CI; P=0.015).  Recurrence-free percentage at 4 years: 73.7% in control vs 89.7% in T arm (HR: 0.47; 95% CI; P<0.001). | Adjuvant chemotherapy with or without trastuzumab |
| **NCCTG-N9831 (63)**  Romond et al, 2005, NEJM. | Gr1: Doxo+Cyclo, then Paclitaxel.  Gr2: Gr1, then T  Gr3: Gr1, T with Paclitaxel |  |  |  |
| **BCIRG-006 (64)**  Slamon et al, 2011, NEJM. | Gr1: Doxo+Cyclo, then Doce  Gr2: Gr1 + T  Gr3: Doce+Carbo  +T, then 34 Weeks T | 3222 patients  Group 1: 1073  Group 2: 1074  Group 3: 1075  Open, phase III, prospective, randomized, multicenter | Primary endpoint: DFS  65 mo Follow-up: 656 events; 5-year DFS in Gr1: 75%; Gr2: 84%; Gr3: 81%; (HR: 0.64; P<0.001). OS 87%, 92%, 91% (HR: 0.63; P<0.001). No difference in efficacy between Gr2 and Gr3. Cardiotoxicity in Gr2 higher than Gr3 (P<0.001). | Adjuvant chemotherapy with or without trastuzumab |
| **FinHer (65)**  Joensuu et al, 2009, J Clin Oncol. | Doce vs. Vinorelbine, followed by FEC in both study arms.  In HER2+ pat, per arm +/- T | 1010 patients  HER2-: 778 Pat  HER2+: 232 Pat  Doce/FEC: 58  Doce/FEC/T: 54  Vino/FEC: 58  Vino/FEC/T: 62  Open, phase III, prospective, randomized, multicenter | Primary endpoint: DDFS  5-year follow-up: 22/115 (19.1%) pat in T arm with distant metastases or death; vs. 31/116 (26.7%) without T (HR: 0.65; 95% CI; 0.38-1.12; P=0.12; where P=0.27 is significant).  Better DFS in terms of distant metastases in the T arm (HR: 0.57; 95% CI; 0.33-0.99; P=0.047). Deaths: 12 pat (10.4%) in the T arm vs. 21 pat (18.1%) in the comparison arm (HR: 0.55; 95% CI, 0.27-1.11; P=0.094). | Adjuvant chemotherapy with or without trastuzumab |
| **APHINITY (53, 66)**  Von Minckwitz et al, 2017, NEJM | P + T + adj ChT vs. T + adj ChT | 2400 patients with P to adj ChT + T vs. 2405 pats with placebo and adj ChT + T.  63% HR+ Pat; 36% HR-negative  Double-blind, phase III, prospective, randomized, multicenter | Primary endpoint: iDFS  Recurrences in 171 patients (7.1%) in the P group; in 210 patients (8.7%) in the placebo group (HR 0.81; 95% CI, 0.66-1.00; P=0.045);  3-year iDFS estimate: 94.1% in P arm; 93.2% in placebo. In HER2+ cohort, 3-year iDFS 92.0% (in P) and 90.2% (placebo). HR (invasive disease event): 0.77; 95% CI, 0.62-0.96; P=0.02; In HRec-negative population, 3-year iDFS 97.5% (in P) and 98.4% (placebo); HR: 1.13; 95% CI, 0.68-1.86; P=0.64. Heart failure, cardiac dysfunction, and cardiovascular death were rare in both groups. Diarrhea grade 3 or higher almost exclusively during ChT and more frequently in the P arm (rather than placebo; 9.8% vs. 3.7%).  6-year iDFS benefit: 4.5% (87.9% vs. 83.4%) in favor of P arm. At longer follow-up, treatment benefit of P not HRec status dependent. | Current standard of care in Austria in the adjuvant setting for nodal-positive tumors. |
| **KATHERINE (9)**  Von Minckwitz et al, 2019, NEJM | Postneoadj T-DM1  vs. T | 1486 randomized patients (743 in the T-DM1 group and 743 in the T group).  Open-label, phase III, prospective, placebo-controlled, randomized, multicenter. | Primary endpoint: iDFS  Invasive disease or death in 91 pat (T-DM1 group, 12.2%) and in 165 pats in the T group (22.2%). 3-year iDFS estimate: 88.3% in the T-DM1 arm; 77.0% in the T arm. HR 0.50; 95% CI; 0.39-0.64; P<0.001. Recurrences in 10.5% of pat (T-DM1) and in 15.9% (T).  Safety data were consistent with previously reported results for T-DM1 therapy, with more adverse events in the T-DM1 arm than in the T arm. | Current standard of care in Austria for residual tumor after neoadjuvant therapy. |
| **ExteNET (12)**  Martin et al, 2017,  Lancet Oncol | Ner vs. placebo after 1 year T | 2840 patients randomized  Ner (n=1420)  Placebo (n=1420)  Double-blind, phase III, prospective, placebo-controlled, randomized, multicenter. | Primary endpoint: iDFS  Significantly fewer iDFS events at 5.2 years (IQR 2.1-5.3) follow-up Ner vs placebo (116 vs 163 events; strat. HR 0.73, 95% CI; 0.57-0.92, P=0.0083). 5-year iDFS: 90.2% (95% CI; 88.3-91.8) with Ner; 87.7% (85.7-89.4) with placebo. Without diarrhea prophylaxis, most common grade 3-4 AEs: diarrhea (561 [40%], grade 3, one pat [<1%] grade 4 on Ner; vs. 23 [2%] grade 3 on placebo), vomiting (grade 3: 47 [3%] vs. five [<1%]), and nausea (grade 3: 26 [2%] vs. two [<1%]).  Treatment-associated SAEs: 103 (7%) pats on Ner; 85 (6%) on placebo. One additional year of therapy with Ner after T+ChT significantly reduced progression or relapse vs. placebo. | Neratinib monotherapy |
|  | | | | |
| **Studies in the neoadjuvant setting** | | | | |
| **NOAH (67)**  Gianni et al,  2010, Lancet | T + neo ChT vs. followed by adj T vs. neo ChT | 117 patients with T vs. 118 with ChT  Open, phase III, prospective, randomized, multicenter | Primary endpoint: EFS  Significant benefit in 3-year EFS in the T group; EFS 71% (95% CI, 61-78; 36 events),  In the ChT arm without T: 56% (46-65; 51 events); HR 0.59 (95% CI, 0.38-0.90; P=0.013).  Despite ChT (doxorubicin) concomitant therapy T well tolerated; Symptomatic heart failure was observed in 2 pat (2%), with good response to cardiac pharmacotherapy. | Trastuzumab neoadjuvant and adjuvant |
| **TRYPHAENA (68)**    Schneeweiss et al, 2013, Ann Oncol | Arm1: Neoadj FEC+T+P, then Doce+T+P  Arm2: Neoadj FEC, then Doce+T+P.  Arm3: Neoadj Doce+Carbo+T+P | 225 patients  Arm1: 73 Pat  Arm2: 75 Pat  Arm3: 77 Pat  Open, phase II, randomized, multicenter | Primary endpoint: cardiac function  Under neoadj treatment: arm2: 2 pat (2.7%) symptomatic left ventricular systolic dysfunction (95% CI); 11 pat (arm1: 4 [5.6%]; arm2: 4 [5.3%]; arm3: 3 [3.9%]; 95% CI)) reduced left ventricular ejection fraction from ≥10% points at baseline to <50%. Most common AE: diarrhea.  pCR (ypT0/is) in 61.6% (arm1), 57.3% (arm2), 66.2% (arm3) of pat. Due to the study design, pCR results are purely descriptive. | Pertuzumab neoadjuvant |
| **NeoSphere (35, 69)**  Gianni et al,  2016, Lancet Oncol | Arm1: Neoadj T+Doce  Arm2: P+T+Doce  Arm3: P+T  Arm4: P+Doce | 417 patients  Arm1: 107 Pat  Arm2: 107 Pat  Arm3: 107 Pat  Arm4: 96 Pat  Open, phase II, randomized, multicenter | Primary endpoint: pCR, (in follow-up publication: PFS and DFS).  Significantly better pCR in 49 of 107 (45.8%) pat in arm2 [95% CI; 36.1 - 55.7]) vs arm1 in 31 of 107 pat; 29.0% [20.6 - 38.5]; P=0.0141).  5-year PFS: arm1: 81% (95% CI; 71-87); arm2: 86% (77-91); arm3: 73% (64-81); arm4: 73% (63-81), (HR: 0.69; 95% CI, arm2 vs. arm1; HR: 1.25; arm3 vs. arm1; HR: 2.05; arm4 vs. arm2).  DFS comparable to PFS results. Safety data and tolerability not significantly different across arms. Most frequent grade ≥3 AEs: neutropenia (arm1: 71 [66%] of 107 pat; arm2: 59 [55%] of 107; arm3: 40 [37%] of 108; arm4: 60 [64%] of 94), leukopenia (arm1: 13 [12%]; arm2: 6 [6%]; arm3: 4 [4%]; arm4: 8 [9%]). | Pertuzumab neoadjuvant - NeoSphere led to approval of combination of trastuzumab and pertuzumab in neoadjuvant setting |
|  | | | | |
| **Studies in the metastatic setting** | | | | |
| **Acronym**  **Author, Year, Journal** | **Substances** | **Patients** | **Results** | **Comments** |
| **CLEOPATRA**  Baselga et al, 2012, NEJM **(4)**  Swain et al, 2019,  J Clin Oncol **(5)** | P + T + Doce vs. T + Doce | 808 patients randomized:  Placebo + T + Doce vs. P + T + Doce  Double-blind, phase III, prospective, placebo-controlled, randomized, multicenter. | Primary endpoint: PFS (independent review).  PFS significantly longer in P group vs placebo: 18.5 vs 12.4 mo (HR for progression or death 0.62; 95% CI; 0.51 - 0.75; P<0.001).  Median OS in favor of P arm at 57.1 mo (402 patients) vs 40.8 mo in placebo arm (406 pat). OS HR 0.69 (95% CI, 0.58; 0.82). 8-year OS 37% (P) vs 23% (placebo).  The OS benefit in previously pretreated T patients in the neo/adj setting was consistent with the overall OS outcome (subgroup = 88 pat, HR 0.86; 95% CI; 0.51 - 1.43).  Safety profile of P + T + Doce therapy was consistent with literature; one additional SAE in P arm indicating heart failure and one symptomatic left ventricular systolic dysfunction; both events resolved without discontinuation of P within 34 days. | First-line standard of care |
| **PERTAIN (44)**  Rimawi et al, 2018,  J Clin Oncol | P + T + AI vs. T + AI | 129 patients per arm; 75 and 71 patients were treated with induction CT, respectively.  Open, phase II, randomized, multicenter | Primary endpoint: PFS  Stratified median PFS in the P+T arm 18.89 mo (95% CI, 14.09 - 27.66 mo) and 15.80 (95% CI, 11.04 - 18.56 mo) in the T arm. Stratified HR: 0.65; 95% CI, 0.48 - 0.89; P =0.0070.  SAEs in 42 (33.1%) of 127 pat in the P arm and in 24 (19.4%) of 124 pat in the T arm. | First-line standard of care in HR+/HER2+ met. Breast cancer in patients not considered candidates for standard treatment |
| **EMILIA**  Verma et al, 2012, NEJM **(8)**  Dieras et al, 2017, Lancet Oncology **(6)** | T-DM1 vs. lap + capecitabine  (After previous T + taxane therapy). | 991 randomized patients  T-DM1 (n=495) or Lap + Cap (control arm: n=496).  Open-label, phase III, randomized, multicenter | Primary endpoint: PFS (independent review), OS, safety.  Median PFS under T-DM1 9.6 mo vs 6.4 mo in the lap + cap arm (HR 0.65; 95% CI; 0.55 - 0.77; P<0.001).  Median OS in favor of T-DM1 at 29.9 mo [95% CI 26.3 - 34.1] vs. control at 25.9 mo [95% CI 22.7 - 28.3]; HR 0.75 [95% CI; 0.64 - 0.88]).  136 (27%) of 496 pat crossed over to the T-DM1 group after the second OS interim analysis (median follow-up 24.1 mo; [IQR 19.5 - 26.1]).  Final OS shows better outcome in favor of T-DM1 over Lap + Cap even after treatment group switch.  Safety profile of T-DM1 was confirmed by previous study analyses: higher incidence of thrombocytopenia and elevated serum aminotransferase with T-DM1; incidences of diarrhea, nausea, vomiting, HFS higher with Lap + Cap therapy | Current second-line standard (and in early recurrence after adjuvant therapy within ≤6 months). |
| **TH3RESA (7)**  Krop et al, 2017, Lancet Oncology. | T-DM1 vs. local SOC (After previous  T + Lap + Taxane therapy and with progression after 2 HER2-targeted regimens). | 602 patients randomized 2:1 to T-DM1 (n=404) or SOC (treatment choice of treating physicians/physicians) (n=198).  Open-label, phase III, randomized, multicenter | Co-primary endpoint: PFS and OS.  Significantly longer OS in T-DM1 arm vs. local SOC (median 22.7 mo [95% CI; 19.4 - 27.5] vs. 15.8 mo [13.5 - 18.7]; HR 0.68 [95% CI; 0.54 - 0.85]; P=0.0007). PFS from interim analysis: longer PFS with T-DM1 therapy vs SOC (median 6.2 mo [95% CI 5.59 - 6.87] vs 3.3 mo [2.89 - 4.14]; HR 0.53 [95% CI; 0.42 - 0.66]; P<0.0001).  93 (47%) of 198 patients from the SOC arm crossed over to T-DM1.  The incidence of grade ≥3 AEs was 161 (40%) of 403 patients in the T-DM1 arm and 87 (47%) of 184 patients in the SOC arm; for SAEs: 102 (25%) of 403 pats with T-DM1 and 41 (22%) of 184 pats in the SOC arm. | In this situation, only rarely used therapeutically as therapy with T-DM1 was usually already given in the second-line setting. |
| **DESTINY Breast01**  Modi et al, 2020, NEJM **(22)**  Modi et al, 2020, SABCS PD3-06 **(48)** | T-DXd (After 6 previous therapies median). | 184 patients  20.1% of patients remained on T-DXd therapy:  80 pats (43.4%) in treatment >12 mo; 11 (6.0%) in treatment >24 mo.  Open-label, phase II, single-arm, multicenter | Primary endpoint: *objective* response (objective response to therapy)  Median follow-up: 11.1 mo (0.7 - 19.9); response to T-DXd in 112 of 184 pat (60.9%; 95% CI; 53.4 - 68.0); median response: 14.8 mo (95% CI; 13.8 - 16.9); median PFS: 16.4 mo (95% CI; 12.7 - not reached). T-DXd shows sustained antitumor activity in heavily pretreated HER2+ metastatic breast cancer pat.  Median follow-up: 20.5 mo (0.7-31.4 mo); Distinctly long duration of response to T-DXd therapy of 20.8 mo; Confirmed response (ORR): 61.4% (95% CI; 54.0%-68.5%). Median PFS: 19.4 mo (95% CI; 14.1-not reached). Robust 18-month OS: 74%. Toxicity and safety profiles along Modi et al. 2020, NEJM. Risk of drug-dependent onset of ILD at 12 mo not associated with cumulative dosing. Sustained benefit/risk profile of T-DXd. | First results with T-DXd |
| **HER2climb (18, 25)**  Murthy et al, 2020, NEJM. | TUC + T + Cap vs. placebo + T + Cap  (After previous therapy in multiple lines with T, P, T-DM1). | PFS in the first 480 patients.  Secondary endpoints evaluated in all patients (n = 612).  Double-blind, phase II, prospective, placebo-controlled, randomized, multicenter. | Primary endpoint: PFS  PFS 1 year: 33.1% with TUC vs 12.3% with placebo (HR: 0.54; 95% CI; 0.42-0.71; P<0.001). Median duration of PFS with TUC: 7.8 mo; with placebo, 5.6 mo.  OS 2 years: 44.9% with TUC vs. 26.6% with placebo + T + cap (HR: 0.66; 95% CI, 0.50 - 0.88; P=0.005); median OS: 21.9 mo (TUC) vs. 17.4 mo (placebo). In pat with brain metastases, 1-year PFS for TUC 24.9% vs 0% for placebo (HR: 0.48; 95% CI; 0.34 - 0.69; P<0.001). Median PFS with TUC 7.6 mo, with placebo 5.4 mo. Common adverse events with TUC: diarrhea, HFS, nausea, fatigue, and vomiting. Diarrhea and elevated aminotransferase levels (≥grade 3) more common with TUC. Better PFS and OS in heavily pretreated pat in metastatic setting with brain metastases with TUC + T + Cap vs. placebo + T + Cap. | Triple combination with tucatinib |
| **NALA (13)**  Saura et al, 2020,  J Clin Oncol | Ner + Cap vs.  Lap + Cap  (After at least 2 previous anti-HER2 therapies). | 621 patients randomized to Ner + Cap n=307; Lap + Cap n=314).  Open-label, phase III, prospective, controlled, randomized, multicenter | Co-primary endpoint: PFS and OS (central review).  PFS improved with Ner addition (HR 0.76; 95% CI; 0.63-0.93; P=0.0059). OS numerically in favor of Ner + Cap, but no significance. HR: 0.88; 95% CI; 0.72 - 1.07; P=0.2098).  Lower intervention for CNS disease needed in Ner arm (cumm. incidence: 22.8% vs 29.2%; P=0.043). ORR 32.8% (Ner; 95% CI; 27.1 - 38.9) and for Lap 26.7% (95% CI; 21.5 - 32.4; P=1201). Median duration on treatment response 8.5 (Ner) vs 5.6 (Lap) mo (HR, 0.50; 95% CI, 0.33 - 0.74; P=0.0004).  Most common AEs: diarrhea (Ner 83% vs. Lap 66%), nausea (53 vs. 42%). Discontinuation of therapy and quality of life score similar in both arms. | First study showing superiority of neratinib over lapatinib |
| **NCT00078572 (10)**  (clinicaltials.gov ID)  Geyer et al, 2006, NEJM. | Lap + Cap vs. Cap | 324 randomized patients  163 Lap + Cap  161 Cap only  Open-label, phase III, prospective, controlled, randomized, multicenter | Primary endpoint: TTP (*time to progression*).  Study showed superiority in the combination therapy group (Lap + Cap), HR for time to relapse 0.49; 95% CI; 0.34 - 0.71; P<0.001), with 49 events in the Lap group vs. 72 events in the Cap monotherapy group.  Median duration to recurrence was 8.4 mo for lap + cap and 4.4 mo for cap.  No increase in cardiac events or severe toxic effects in the combination group. | Randomized trial capecitabine plus/minus lapatinib, led to approval of the combination of capecitabine and lapatinib |
| **HERMINE (36)**  Extra et al, 2010, Oncologist | T and TBP | 623 patients observed over 2 years;  Subgroup TBP 177 patients  Non-interventional, cohort, prospective and retrospective. | Primary endpoint: treatment duration with T  Median treatment duration of 13.3 mo; In 1st, 2nd, and 3rd line (or 4+), median TTP was 10.3; 9.9; and 6.3 mo; median OS corresponded to 30.3; 27.1; and 23.2 mo, respectively. Cardiac failure was observed in 2.6% of pat.  In the TBP subgroup analysis, it was observed:  median OS since initiation of therapy and duration to progression were both longer in the TBP group than in the comparison group that discontinued T (>27.8 vs. 21.3 mo; and 16.8 vs. 4.6 mo, respectively).  groups were not entirely comparable, as the TBP group had a better prognosis at baseline. Median TTP was 10.2 mo for TBP vs. 7.1 for the group that discontinued T. | Data from numerous studies suggest that even in cases of progression on HER2-targeted therapy, continuing this anti-HER2 treatment leads to better outcomes. |
| **GBG 26 / BIG 3-05**  Von Minckwitz et al, 2009, J Clin Oncol **(38)**  Von Minckwitz et al, 2011, E J Cancer.  **(39)** | Cap vs. Cap + T | Patients randomized to cap (n=78);  Cap + T (N=78)  Further follow-up in 74 cap patients and 77 cap + T patients.  Open-label, phase III, prospective, controlled, randomized, multicenter | Primary endpoint: TTP  Median TTP 5.6 mo (cap), 8.2 mo (cap + T); HR 0.69 (95% CI, 0.48-0.97; P=0.0338). 15.6 Mo Follow-Up: 65 events, 38 deaths in CAP arm vs. 62 events, 33 deaths in Cap + T arm. OS: 20.4 mo (95% CI, 17.8 - 24.7) cap; 25.5 mo (95% CI, 19.0 - 30.7) cap + T (P=0.257). ORR: 27.0% cap; 48.1% cap + T (odds ratio 2.5; P=0.0115). No significant increase in toxicity with TBP.  20.7 mo follow-up: 59/74 deaths (cap); 60/77 cap + T. Median OS: 20.6 mo cap and 24.9 mo cap + T; (HR=0.94 [0.65 - 1.35]; P=0.73). No difference between arms in clinical response or success. For continuation/resumption of anti-HER2 treatment after second progression (T or Lap), survival after progression of 18.8 vs 13.3 mo in favor of pat with continuation of therapy (HR 0.63; P=0.02). | Data from numerous studies suggest that even in cases of progression on trastuzumab-based therapy, continuing trastuzumab treatment leads to better outcomes. |
| **PRECIOUS (70)**  Yamamoto et al, 2020, SABCS PD3-11. | P + T + ChT vs.  T + ChT in the 3rd/4th line | 217 patients randomized  108 P + T + ChT  109 T + ChT  Open-label, phase III, prospective, controlled, randomized, multicenter | Primary endpoint: PFS (*investigator-assessed*).  PFS and OS events: 184 (84.8%) in the P + T + ChT group; 84 (38.7%) in the T + ChT group.  Median follow-up: 14.2 mo. PFS significantly better in P arm (median PFS 5.3 vs. 4.2 mo; HR = 0.755; 95% CI; upper limit 0.967; P=0.0217).  Median PFS after T-DM1 therapy 5.3 vs 4.2 mo (HR = 0.801; 95% CI; upper limit 1.061; P=0.0952) and OS (28.8 vs 23.4 mo; HR = 0.713; 95% CI; upper limit 1.026; P=0.062) appeared longer in the P group-further follow-up is needed.  There were no significant group differences in ORR, SAEs, and safety data. | The interim analysis shows that pertuzumab may still be an option in later lines, even if it has been used in earlier lines. |
| **monarcHER (42)**  Tolaney et al, 2020, Lancet Oncology. | Abe+T+Ful (A), or Abe+T (B), or SOC [ChT+T] (C) | 237 patients were randomized 1:1:1:  Abe+T+Ful (A=79),  Abe+T (B=79), or  SOC [ChT+T] (C=79)  Open-label, phase II, prospective, controlled, randomized, multicenter | Primary endpoint: PFS (*investigator-assessed*).  Median follow-up of 19.0 mo. Median PFS between group A (8.3 mo, 95% CI 5.9-12.6) and group C (5.7 mo, 5.4-7.0; HR 0.67 [95% CI 0.45-1.00]; P=0.051) significantly in favor of group A. No PFS difference between group B and C.  The most common treatment-associated grade 3-4 AE in groups A, B, C was neutropenia with (21 [27%] of 78 pats, 17 [22%] of 77 pats, and 19 [26%] of 72).  Most common treatment-associated SAEs:  Group A: Pyrexia (3 [4%]), diarrhea (2 [3%]), urinary tract infection (2 [3%]), and acute renal failure (2 [3%]);  Group B: diarrhea (2 [3%]) and pneumonitis (2 [3%]);  Group C: Neutropenia (4 [6%]) and pleural effusion (2 [3%]). | Treatment option Abe+T+Ful in pretreated patients with HR+/ HER2+ metastatic breast cancer. *Caution*: No approval! |
| **PANACEA (43)**  Loi et al, 2019, Lancet Oncology | Pem+T | 6 patients in phase 1b in dose escalation from Pem;  In phase 2, 52 pats (40 with PD-L1+; 12 with PD-L1- tumor).  Single-arm, phase Ib/II, multicenter, | Primary endpoint: response of PD-L1+ patients.  Median follow-up in phase 2: 13.6 mo (IQR 11.6-18.4) For PD-L1-positive pats; and 12.2 mo (7.9-12.2) for pats with PD-L1-negative tumors.  6 of the 40 PD-L1+ pats (15%, 90% CI, 7-29) showed positive response. No response in the PD-L1- cohort. Most common treatment-associated AE was fatigue in 12 of 58 (21%) pats.  Grade 3-5 AEs were observed in 50% (29) of pats.  Treatment-associated AEs of grades 3-5 were described in 29% (17) of pats.  The most common SAEs: dyspnea (n=3 [5%]), pneumonitis (n=3 [5%]), pericardial effusion (n=2 [3%]), and upper respiratory tract infection (n=2 [3%]).  One treatment-associated death due to Lambert-Eaton syndrome in a PD-L1-negative patient has been described. | Potential clinical benefit of combining trastuzumab and pembrolizumab in pretreated patients with metastatic (PD-L1 positive) disease. Caveat: *No approval!* |
| **EGF104900 (40)**  Blackwell et al, 2010,  J Clin Oncol | L vs. L + T | 296 patients after a median of 3 previous T cycles of therapy.  Open-label, phase III, prospective, controlled, randomized, multicenter | Primary endpoint: PFS  Significantly higher PFS (HR = 0.73; 95% CI, 0.57-0.93; P=0.008) and clinical benefit (24.7%) in the combination arm (L+T) vs. 12.4% with L (P=0.01).  No distinction in ORR.  A trend in favor of L+T therapy for OS (HR = 0.75; 95% CI, 0.53 - 1.07; P=0.106).  Most common AEs: diarrhea, rash, nausea, and fatigue.  Diarrhea was more pronounced in the combination arm (P=0.03).  The incidence of symptomatic and asymptomatic cardiac events was low (L+T: 2.0 and 3.4%; L: 0.7 and 1.4%). | Superiority of vertical dual blockade of trastuzumab and lapatinib versus lapatinib alone in pretreated patients. |
| **TAnDEM (71)**  Kaufman et al, 2009,  J Clin Oncol | T + Ana vs. | 103 patients with T + Ana therapy.  104 with Ana monotherapy  Open-label, phase III, prospective, controlled, randomized, multicenter | Primary endpoint: PFS  PFS with T+ana significantly better than with ana monotherapy (HR = 0.63; 95% CI, 0.47 - 0.84; median PFS, 4.8 vs. 2.4 months; log-rank P=0.0016).  In HRec+ pats (n = 150) median PFS 5.6 (T+Ana) and 3.8 mo in Ana (P=0.006).  Differences in OS in the overall population and the HRec+ population showed no statistical significance.  70% of pats (Ana arm) switched to the T+Ana arm after progression.  Incidences of grade 3 and 4 AEs in the T+Ana arm: 23 and 5%; in the Ana arm: 15 and 1%.  One patient in the combination group showed NYHA II heart failure. | First phase III study of hormone therapy plus T in HER2+, HR+ patients with metastatic breast cancer. |
| **EGF30008 (11)**  Johnston et al, 2009,  J Clin Oncol | Letro+Lap vs. Letro+Placebo | 219 HRec+, HER2+ patients  Double-blind, phase III, prospective, controlled, randomized, multicenter. | Primary endpoint: PFS  Significant risk reduction of progression with Lap+Letro therapy vs. Letro+Placebo; median PFS in favor of Lap 8.2 vs. 3.0 mo. (HR = 0.71; 95% CI; 0.53 - 0.96; P=0.019);  Clinical benefit (treatment response or stable disease ≥6 mo) was significantly better in the Lap+Letro group vs. Letro+Placebo (48% vs. 29%; odds ratio [OR] = 0.4; 95% CI, 0.2-0.8; P=0.003). | Lap + letrozole as first-line therapy in HR+/HER2+ metastatic breast cancer. |
| **ALTERNATIVE (41)**  Johnston et al, 2021, J Clin Oncol | Lap+T+AI  T+AI  Lap+AI | 355 patients randomized into 3 arms:  Lap+T+AI (n=120)  T+AI (n=117)  Lap+AI (n=118)  Open-label, phase III, prospective, controlled, randomized, multicenter | Primary endpoint: PFS  Superiority in terms of PFS in Lap+T+AI group vs. T+AI (median PFS 11.0 vs. 5.6 mo; HR 0.62 [95% CI, 0.45-0.88]; P=0.0063).  ORR, CBR, and OS equally improved in the Lap+T+AI group.  Median PFS with Lap+AI vs T+AI was 8.3 vs 5.6 mo (HR 0.85 [95% CI; 0.62 - 1.17]; P=0.3159).  The most common grade 1-2 AEs (≥ 15%) in each group, Lap+T+AI, T+AI, Lap+AI:  Diarrhea: (69%, 9%, and 51%,), rash (36%, 2%, and 28%), nausea (22%, 9%, and 22%), and paronychia (30%, 0%, and 15%).  SAEs comparable in all 3 groups. | Efficacy and safety of dual HER2 blockade plus AI in HR+/HER2+ metastatic breast cancer. |

**Supplementary Table 2:** Overview of selected studies in the neo-/adjuvant or metastatic setting.

P = pertuzumab; T = trastuzumab; T-DM1 = trastuzumab emtansine; Doce = docetaxel; Lap = lapatinib; Ner = neratinib; T-DXd = trastuzumab deruxtecan; Cap = capecitabine; TUC = tucatinib; Ana = anastrozole; Letro = letrozole; AI = aromatase inhibitor; Abe = abemaciclib; Ful = fulvestrant; Pem = pembrolizumab; Doxo = doxorubicin; Cyclo = cyclophosphamide; Carbo = carboplatin; FEC = fluorouracil, epirubicin, cyclophosphamide; ChT = chemotherapy; adj = adjuvant; neo = neoadjuvant; HR = hazard ratio; CI = confidence interval; DFS = disease-free survival; OS = overall survival; HRec = hormone receptor; iDFS = invasive DFS; PFS = progress-free survival; AZ = general condition; SAE = serious adverse event; pCR = pathological complete response; LVEF = left ventricular ejection fraction; HFS = hand-foot syndrome; IQR = interquartile range; SOC = standard of care/treatment choice of local center/investigator; ORR = objective response rate [objective response + partial response to therapy]; ILD = interstitial lung disease; CNS = central nervous system; TBP = trastuzumab beyond progression; TTP = time to progression; NYHA = New York Heart Association; CBR = clinical benefit ratio.
